# Enzymes in the glutamate-glutamine cycle in the anterior cingulate cortex in postmortem brain of subjects with autism

**DOI:** 10.1186/2040-2392-4-6

**Published:** 2013-03-26

**Authors:** Chie Shimmura, Katsuaki Suzuki, Yasuhide Iwata, Kenji J Tsuchiya, Koji Ohno, Hideo Matsuzaki, Keiko Iwata, Yosuke Kameno, Taro Takahashi, Tomoyasu Wakuda, Kazuhiko Nakamura, Kenji Hashimoto, Norio Mori

**Affiliations:** 1 Research Center for Child Mental Development, Hamamatsu University School of Medicine, Hamamatsu, Japan; 2 Department of Psychiatry, Hamamatsu University School of Medicine, Hamamatsu, Japan; 3 Department of Anatomy and Neuroscience, Hamamatsu University School of Medicine, Hamamatsu, Japan; 4 Research Center for Child Mental Development, University of Fukui, Fukui, Japan; 5 Center of Forensic Mental Health, Chiba University, Chiba, Japan

**Keywords:** Autism, Glutamate, Glutaminase, Glutamate-glutamine cycle, Anterior cingulate cortex

## Abstract

**Background:**

Accumulating evidence suggests that dysfunction in the glutamatergic system may underlie the pathophysiology of autism. The anterior cingulate cortex (ACC) has been implicated in autism as well as in glutamatergic neurotransmission. We hypothesized that alterations in the glutamate-glutamine cycle in the ACC might play a role in the pathophysiology of autism.

**Methods:**

We performed Western blot analyses for the protein expression levels of enzymes in the glutamate-glutamine cycle, including glutamine synthetase, kidney-type glutaminase, liver-type glutaminase, and glutamate dehydrogenases 1 and 2, in the ACC of postmortem brain of individuals with autism (n = 7) and control subjects (n = 13).

**Results:**

We found that the protein levels of kidney-type glutaminase, but not those of the other enzymes measured, in the ACC were significantly lower in subjects with autism than in controls.

**Conclusion:**

The results suggest that reduced expression of kidney-type glutaminase may account for putative alterations in glutamatergic neurotransmission in the ACC in autism.

## Background

Autism is a neurodevelopmental disorder that is characterized by severe and sustained impairment in social interaction, deficits in verbal and nonverbal communication, and repetitive and restricted patterns of behavior and interests [1]. One region that has been implicated in the pathophysiology of autism is the anterior cingulate cortex (ACC), an area crucial for social cognitive processes [2-4]. Neuroimaging studies using magnetic resonance imaging and/or positron emission tomography have suggested structural and functional alterations in the ACC of people with autism [5-8]. For instance, Haznedar and colleagues [9] reported that the ACC was significantly smaller in relative volume, and metabolically less active, in individuals with autism than in normal subjects. Furthermore, a meta-analysis of functional neuroimaging studies for autism and related disorders, namely, autism spectrum disorders (ASDs), revealed that subjects with ASD showed a greater likelihood of hypoactivation in the ACC compared with that of controls [10]. It is speculated that at least some of these findings are attributable to abnormalities in glutamatergic neurotransmission, because both efferents from the ACC to the ventral striatum and afferents from the thalamus to ACC neurons are mediated by a neurotransmitter, glutamate [11]. Postmortem studies have suggested that dysfunction in the glutamatergic system in the cerebellum may be involved in the pathophysiology of autism [12]. For instance, Purcell and colleagues [13] reported that the mRNA levels of genes, including those for the excitatory amino acid transporter 1 and AMPA-type glutamate receptor, are significantly increased in the postmortem cerebellum of autistic individuals, while AMPA-type glutamate receptor density is somewhat decreased in people with autism. Thus, extracellular glutamate levels in the cerebellum of people with autism may be extremely high [14,15]. However, it remains unclear whether or not a similar profile applies to the ACC of individuals with autism. In a proton magnetic resonance spectroscopy (^1^H-MRS) study, Bernardi and colleagues [16] reported a reduction in Glx, a measure of the mixture of glutamate and glutamine, in the right ACC of adults with ASD, although another ^1^H-MRS study revealed no such difference in Glx in the ACC of children with ASD [17]. Such inconsistent results might be attributable to differences in the age range of the subjects studied and/or in the resolution of the equipment used.

Glutamate is synthesized from glutamine by glutaminase in neurons of the brain. After the glutamate is released from the synaptic terminal, it is taken up into astrocytes, where it is converted into glutamine by glutamine synthetase; the glutamine is then transported to the neurons and reused. This ‘glutamate-glutamine cycle’ is an important constituent of the glutamatergic neurotransmission system. On the basis of these findings, we hypothesized that the glutamate-glutamine cycle is impaired in the brains of autistic individuals, and that the enzymes associated with this cycle are dysregulated. To probe for direct evidence of any differences in the levels of glutamate-glutamine cycle-related enzymes between the brain of individuals with autism and that of healthy subjects, we examined here the protein levels of the following enzymes associated with the glutamate-glutamine cycle in the postmortem brains of individuals with autism: glutamine synthetase (GS), which converts glutamate to glutamine; kidney-type glutaminase (KGA), which synthesizes glutamate from glutamine; liver-type glutaminase (LGA), which is an isozyme dominant in the liver; and glutamate dehydrogenases 1 (GDH1) and 2 (GDH2), both of which synthesize glutamate from α-ketoglutarate. We also examined free glutamate and glutamine levels in the ACC of postmortem brains of individuals with autism.

## Methods

### Postmortem brain tissues

The ethics committee of the Hamamatsu University School of Medicine approved this study, in which all experimental procedures were carried out in compliance with the Declaration of Helsinki. Postmortem cingulate blocks were obtained from the Autism Tissue Program (Princeton, NJ, USA) and the NICHD Brain and Tissue Bank for Developmental Disorders (Baltimore, MD, USA) (n = 7 autistic brain samples from six males and a female; n = 13 control brain samples from nine males and four females). The demographic characteristics are shown in Table 1. We have chosen samples from donors with relatively wide age range, from children to adults, in order to examine possible effects of age on the levels of expression of enzymes as well as concentrations of amino acids. Samples were matched for age, gender, and postmortem interval (PMI). Autism was diagnosed according to the DSM-IV-TR, and the diagnosis was confirmed by the Autism Diagnostic Interview - revised. All samples were stored at -80°C until use. Information obtained from the next of kin showed that none of the controls had any known history of neuropsychiatric disorders or illicit drug use.

**Table 1:** Demographic variables for control subjects and individuals with autism

**Case number**	**Disorder**	**Age (years)**	**Gender**	**PMI (hours)**	**Cause of death**	**History of seizures**
C01	No known disorder	27	M	10	Multiple injuries	No
C02	No known disorder	15	M	12	N/A	No
C03	No known disorder	15	M	16	Multiple injuries	No
C04	No known disorder	9	F	20	Asthma	No
C05	No known disorder	20	F	19	Head injuries	No
C06	No known disorder	20	M	22	Multiple injuries	No
C07	No known disorder	8	F	20	Traumatic multiple injury	No
C08	No known disorder	14	M	18	Multiple injuries	No
C09	No known disorder	12	M	19	Drowning	No
C10	No known disorder	8	M	5	Cardiac arrhythmia	No
C11	No known disorder	29	M	13	Traumatic multiple injuries	No
C12	No known disorder	15	F	5	Chest injuries	No
C13	No known disorder	14	M	16	Multiple injuries	No
**Control (n = 13)**		**15.8 ± 6.6**	**M:F = 9:4**	**15.0 ± 5.6**		
A01	Autism	9	M	13	Drowning	No
A02	Autism	8	M	12	Drowning	No
A03	Autism	8	M	16	Drowning	No
A04	Autism	14	M	9	Drowning	No
A05	Autism	27	M	8.3	Drowning	No
A06	Autism	16	M	N/A	Seizures	Yes
A07	Autism	29	F	17.83	Seizures	Yes
**Autism (n = 7)**		**15.9 ± 8.9**	**M:F = 6:1**	**12.7 ± 3.8**		

Values are shown as means ± standard deviation.

Abbreviations: F, female; M, male; N/A, not available; PMI, postmortem interval.

### Western blotting

Brain tissue (approximately 50 mg) from each subject was cut and placed in ice-cold RIPA buffer containing protease inhibitor cocktail (Complete Protease Inhibitor Cocktail Tablets; Roche, Basel, Switzerland). Tissue samples were homogenized by sonication while the temperature was maintained at 4°C. The homogenates were centrifuged at 10,000 × g for 20 minutes at 4°C. The supernatants were collected, and the total protein content was assayed using the BCA protein assay (Thermo Scientific, Waltham, MA, USA).

Ten micrograms of each brain lysate was loaded onto a 7.5% SDS-PAGE. The proteins were electroblotted onto PVF membrane (Millipore, Billerica, MA, USA). Protein blots were washed and blocked for one hour at room temperature with 1% nonfat dry milk in tris-buffered saline (pH = 7.4). The following proteins were analyzed: GS, KGA, LGA, GDH1, GDH2, glial fibrillary acidic protein (GFAP) as a marker of astrocyte [18], neuron-specific enolase (NSE) as a marker of neuronal integrity [19], and ß-actin served as an internal control. The blots were incubated with primary antibody overnight at 4°C (anti-GS, 1: 2000, from Abcam, (Cambridge, UK); anti-KGA; 1: 1000, from Protein Tech Group Inc. (Chicago, IL, USA); anti-LGA, 1: 400, from Aviva Systems Biology (San Diego, CA, USA); anti-GDH1, 1: 1000, from Abcam (Cambridge, UK); anti-GDH2, 1: 500, from Abnova (Taipei, Taiwan); anti-GFAP, 1: 4000, from Chemicon International (Temecula, CA, USA); anti-NSE, 1: 2000 and anti-ß-actin, 1: 2000, both from Abcam (Cambridge, UK), followed by horseradish peroxidase-conjugated secondary antibody incubation for one hour at room temperature. The immune complexes were visualized using the ECL Plus detection system (GE Healthcare, Buckinghamshire, UK) and were exposed to Fuji medical x-ray film (Fuji Photo Film, Tokyo, Japan).

### Measurement of amino acids

An examiner who was blinded to the diagnosis used high-performance liquid chromatography (HPLC) to measure free glutamate and glutamine in postmortem brain tissues. The brain tissues were homogenized in 20 volumes of methanol (HPLC grade) on ice. The homogenates were centrifuged at 4,500 × *g* for 10 minutes, and 20 μL of supernatant was evaporated to dryness at 40°C. To the residue, 20 μL of water (H_2_O) (HPLC grade), 20 μL of 0.1 mol/L borate buffer (pH = 8.0), and 60 μL of 50 mmol/L 4-fluoro-7-nitro-2,1,3-benzoxadiazole (Tokyo Kasei Kogyo Co. Ltd., Tokyo, Japan) in acetonitrile (CH_3_CN) (HPLC grade) were added. The reaction mixture was then heated at 60°C for one min and immediately supplemented with 100 μL of H_2_O/CH_3_CN (90/10) containing 0.1% trifluoroacetic acid to stop the reaction. The resultant solution was injected into the HPLC system in a volume of 10 μL.

### Statistical analysis

Each expression value of the enzymes using Western blotting was normalized by the ß-actin level. The Mann–Whitney *U* test was employed, and a two-tailed *P*-value was established to test for significance. The gender difference between the two groups was examined by Fisher's exact test. The relationships among the enzyme expression values and the relationships between enzyme expression values and age or PMI were evaluated by Spearman’s ρ correlation coefficients. The between-group differences in the level of each amino acid were analyzed using the Mann–Whitney *U* test. Data are expressed as mean ± standard deviation. Statistical significance was set at *P* < 0.05.

## Results

With regard to the postmortem brain tissues, there was no significant difference in age (*U* = 42.5, *P* = 0.84), PMI (*U* = 25.0, *P* = 0.23), or gender (*P* = 0.61) between the autistic and control groups (Table 1). With respect to the cause of death, five of the seven deaths of autistic subjects were due to drowning. Among the controls, nine of thirteen deaths were due to traumatic injury and only one death was due to drowning. Two of the autistic subjects had a history of epilepsy.

**Figure 1:**
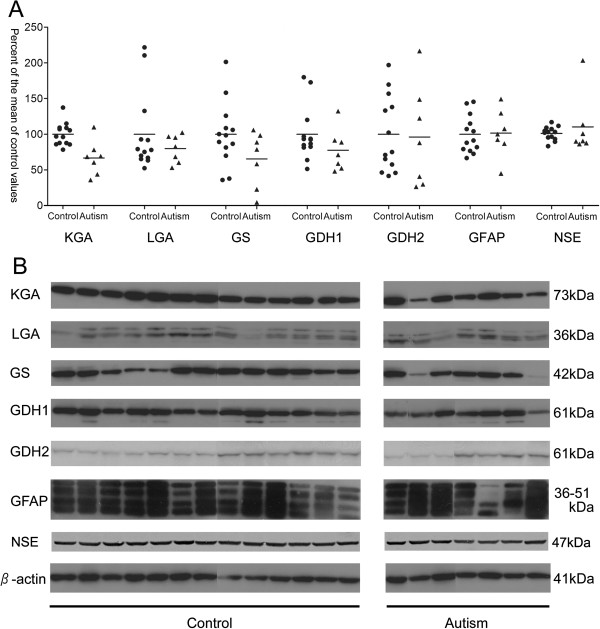
**Expression levels of enzymes associated with the glutamate-glutamine cycle. (A)** Scatter plots of enzyme optical density values for control subjects (n = 13) and individuals with autism (n = 7). Results are expressed as a percent of the control values, normalized by the β-actin level. **(B) **Western blots showing immunolabeling of KGA, LGA, GS, GDH1, GDH2, GFAP, NSE, and β-actin in the anterior cingulate cortex (ACC) of control subjects (n = 13) and individuals with autism (n = 7). Abbreviations: GDH1, glutamate dehydrogenase 1; GDH2, glutamate dehydrogenase 2; GFAP, glial fibrillary acidic protein; GS, glutamine synthetase; KGA, kidney-type glutaminase; LGA, liver-type glutaminase; NSE, neuron-specific enolase.

There were significant differences in the expression levels of only one of the five enzymes, KGA, between the autistic and control groups (*U* = 11.0, *P* = 0.007, Figure 1). The mean KGA value was 34% lower in the autistic group than in the control group. The KGA level did not correlate with age or with PMI. The concentrations of the remaining five proteins examined did not differ between the two groups: GS, *U* = 24.0, *P* = 0.10; LGA, *U* = 44.0, *P* = 0.93; GDH1, *U* = 27.0, *P* = 0.15; GDH2, *U* = 38.0, *P* = 0.58; GFAP, *U* = 39.0, *P* = 0.63; and NSE, *U* = 36.0, *P* = 0.48 (Figure 1). There was no statistically significant correlation between age of donors and levels of LGA, GS, GDH1, GDH2, GFAP, or NSE (Table 2 and Additional file 1). According to the record of samples, case A7 received medication with sodium valproate and topiramate. These anticonvulsants may have affected the results. When case A7 was removed from the analysis, the difference in KGA levels remained significant between subjects with autism and controls (*U* = 11.0, *P* = 0.015).

**Table 2:** Results from correlation analyses between age of donors and expression levels of proteins and amino acids

		**KGA**	**LGA**	**GS**	**GDH1**	**GDH2**	**GFAP**	**NSE**	**Glu**	**Gln**	**Glu/Gln**
Total sample (n = 20)	Pearson r correlation coefficient	-.14	.10	-.13	-.04	.20	-.25	.22	.09	.35	-.32
	*P-*value	.56	.68	.58	.87	.39	.29	.35	.72	.13	.17
Control (n = 13)	Pearson r correlation coefficient	-.24	.00	-.30	-.07	-.07	-.44	.37	.34	.54	-.48
	*P-*value	.42	.98	.32	.81	.80	.12	.21	.25	.06	.09
Autism (n = 7)	Pearson r correlation coefficient	.22	-.13	-.05	.05	.29	.14	.56	-.05	.07	-.16
	*P*-value	.66	.78	.91	.90	.56	.78	.20	.90	.90	.71

Abbreviations: GDH1, glutamate dehydrogenase 1; GDH2, glutamate dehydrogenase 2; GFAP, glial fibrillary acidic protein; Gln, glutamine; Glu, glutamate; GS, glutamine synthetase; KGA, kidney-type glutaminase; LGA, liver-type glutaminase; NSE, neuron-specific enolase.

**Figure 2:**
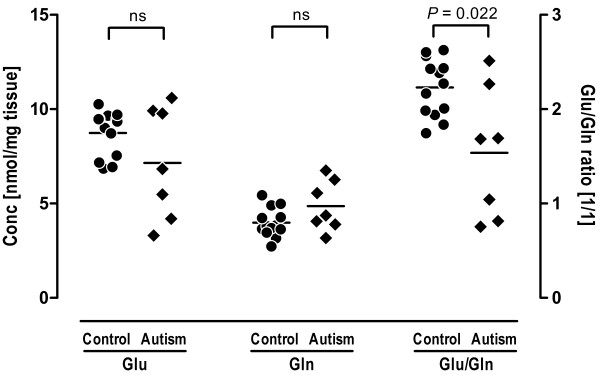
**Free glutamate and glutamine concentrations in the ACC from brains of control and autistic subjects. **There were no significant differences in either glutamate or glutamine concentrations between the two groups. The ratio of glutamate to glutamine (Glu/Gln) was significantly lower in the autistic subjects than in controls (*U* = 16.0, *P = 0.022). Abbreviations: Conc, concentration; Gln, glutamine; Glu, glutamate; ns, not significant.

The tissue levels of glutamate and glutamine are shown in Figure 2. There were no significant differences between the two groups in free glutamate (7.8 ± 2.4 nmol/mg tissue in the autistic group versus 8.7 ± 1.2 nmol/mg tissue in the control group, *U* = 41.0, *P* = 0.75) or glutamine levels (4.8 ± 1.4 nmol/mg tissue in the autistic group versus 4.0 ± 0.8 nmol/mg tissue in the control group, *U* = 25.0, *P* = 0.11). However, the ratio of glutamate to glutamine (Glu/Gln ratio) was significantly smaller in the autistic tissue than in the controls (1.54 ± 0.70 in the autistic group versus 2.23 ± 0.30 in the control group, *U* = 16.0, *P* = 0.022). There was no statistically significant correlation between age and levels of glutamate, glutamine, or Glu/Gln ratio (Table 2 and Additional file 2).

## Discussion

In the present study, we found that KGA expression in the ACC of postmortem brain tissue was significantly lower in individuals with autism than in controls. In contrast, no changes were observed in the other enzymes examined; that is, LGA, GS, GDH1, and GDH2 levels were the same in autism and control subjects. Furthermore, protein levels of either GFAP or NSE were not different between the two groups. These results suggest that KGA expression may be selectively reduced among enzymes participating in the glutamate-glutamine cycle in the ACC in individuals with autism.

The quality of proteins isolated from the postmortem brain depends on the PMI and on the agonal state preceding death by causes such as hypoxia or other trauma to the brain [20]. It has been reported that PMI had no effect on glutaminase levels in the postmortem brain [21,22]. Given that there was no significant difference in PMI between the two groups in the current study, it is unlikely that the PMI influenced our results. With regard to the cause of death, five of the seven subjects with autism died by drowning, while the cause of mortality in most of the control cases was traumatic injury. It is possible that the individuals who died from drowning had been exposed to an ischemic or hypoxic state for a longer duration than were those who died due to traumatic injury. This, in turn, could have affected the KGA expression levels. To the best of our knowledge, there are no reports of specific KGA reductions in tissue in an ischemic or hypoxic state. Further studies are needed in this regard. It is also important to consider the presence or absence of a history of seizures, because glutamate plays an important role in the initiation and propagation of epileptic seizures [23]. It has been reported that patients with mesial temporal lobe epilepsy have increased basal concentrations of extracellular glutamate in the epileptogenic hippocampus [24]. Eid and colleagues [25] have shown that protein levels of phosphate-activated glutaminase (that is, KGA) in hippocampal homogenates were not significantly different between patients with mesial temporal lobe epilepsy and patients with other forms of temporal lobe epilepsy (non-epileptogenic hippocampus). However, no information is currently available regarding whether or not the expression of glutaminase in the ACC is altered in patients with epilepsy. In our postmortem brain samples, A6 and A7 had a history of epilepsy; KGA expression in A6 was a little higher than the mean level in the autism group, whereas that in A7 was lower. However, neither case was an outlier. Therefore, it is unlikely that the history of epilepsy in these two cases contributed to our results. With regard to the data for GDH2, very low levels of expression were observed in three samples from autism while the remaining four samples showed medium-high levels of expression. Since the variance in GDH2 levels did not differ significantly between the two groups, it is unclear from this preliminary study whether the distribution of GDH2 levels in autism represents two possible subgroups.

The findings of the present study suggested that KGA, which converts glutamine into glutamate, is significantly reduced in the ACC of individuals with autism. The KGA isozyme, which is ubiquitous in all tissues, is expressed predominantly in the mitochondria of neurons in the brain [26]. In addition, no significant differences were observed between the two groups in terms of the expression levels of NSE, which served as markers for the number of neurons. Therefore, our results suggest that the expression of KGA is altered at the protein level in the ACC neurons in subjects with autism. The mechanism by which KGA expression is reduced in the ACC is unknown. To the best of our knowledge, this is the first demonstration of KGA expression in the ACC of postmortem brain from individuals with autism. One possible explanation is that the reduction in the expression of KGA isozyme may represent an altered function of glutamatergic neurons in the ACC in subjects with autism. This view could be supported by results from measurements of amino acids in the present study. Although the differences in free glutamate and glutamine concentrations between the two groups did not reach statistical significance, the Glu/Gln ratio was lower in autistic samples compared with controls. This suggests that the glutamate-glutamine cycle in the ACC may shift toward glutamine rather than glutamate in autism. These findings may provide further evidence of impaired glutamatergic transmission, which was previously implicated in the pathophysiology of autism [27].

Glutamine is a precursor of glutamate, which is further converted into γ-aminobutyric acid (GABA) by glutamate decarboxylase in GABAergic neurons. KGA exists in both glutamatergic and GABAergic neurons and is involved in the production of both neurotransmitters [28]. Therefore, the reduction of KGA expression in our study might have been the result of alterations not only in glutamatergic but also in GABAergic neurons. In fact, the density of both GABA_A_ and GABA_B_ receptors was decreased in the ACC of individuals with autism [29,30], suggesting the dysfunction of the GABAergic projection or GABA release in the ACC.

Neurons in the ACC project to the ventral striatum, from which projections pass to the globus pallidus, ventral pallidum, and substantia nigra, the subcortical structures known to modulate cortical information processing by different neurotransmitter systems [2,11]. The present study found a significant reduction in KGA in the ACC, suggesting that excitatory neurotransmissions through the ACC would be impaired in autism. The abnormal connectivity passing through the ACC and related regions can increase the ratio of excitation/inhibition of a wide variety of brain regions, which is postulated to be the common pathway for causing autism [31].

The LGA isozyme was originally thought to be present only in the liver [32]. Recently, LGA was found to be expressed in astrocytes in the brain, although its functional role remains to be elucidated [33]. In our study, the expression levels of LGA, GS, GDH1, and GDH2, all of which are expressed in astrocytes, were similar in the autism and control groups. In addition, no significant differences were observed between the two groups in terms of GFAP expression levels in the ACC. This finding was consistent with those of a previous report [34] showing increased expression of GFAP in the cerebellum, but not in the ACC, in the postmortem brain of individuals with autism.

The lack of a significant change in tissue levels of glutamate or glutamine suggests that the regional contents of glutamate and glutamine in the ACC may not be altered in people with autism. The fact that the tissue levels of glutamate and glutamine remained constant is of little importance in this respect, since the tissue level does not reflect the turnover of the pool of each amino acid. Indeed, Friedman *et al*. [17] revealed in a ^1^H-MRS study that levels of Glx in the ACC did not differ between children with ASD and typically-developed controls. In contrast, DeVito *et al*. [5] reported finding widespread Glx reductions in the gray matter of children with ASD. Using 3T ^1^H-MRS, Bernardi *et al*. [16] demonstrated that adults with ASD showed significantly lower Glx concentration in the right, but not the left, ACC compared to controls. Since the samples used in the present study were all from the left ACC, our results could be considered consistent with those of Friedman *et al*. [17] and Bernardi *et al*. [16]. Given that there was no concomitant reduction in other enzymes in the glutamate-glutamine cycle, most of which are essentially expressed in astrocytes, the negative result of the tissue levels of these two amino acids may be attributable to the compensatory function of astrocytes.

## Conclusion

In conclusion, this study provides evidence of a reduction in KGA expression in the ACC of the postmortem brain of individuals with autism. The finding indicates a dysfunction in excitatory amino acid neurotransmission in this brain region in the disorder. Since the small sample size renders the data presented here preliminary, studies with more subjects with ASD that examine more regions of the brain than the ACC are necessary.

## Abbreviations

ACC: anterior cingulate cortex; AMPA: α-amino-3-hydroxy-5-methyl-4-isoxazolepropionic acid; ASD: autism spectrum disorder; DSM-IV-TR: Diagnostic Statistical Manual for mental disorders fourth edition text revision; GABA: γ-aminobutyric acid; GDH1: glutamate dehydrogenase 1; GDH2: glutamate dehydrogenase 2; GFAP: glial fibrillary acidic protein; Gln: glutamine; Glu: glutamate; Glx: a measure of the mixture of glutamate and glutamine; GS: glutamine synthetase; 1H-MRS: proton magnetic resonance spectroscopy; HPLC: high-performance liquid chromatography; KGA: kidney-type glutaminase; LGA: liver-type glutaminase; NSE: neuron-specific enolase; PMI: postmortem interval.

## Competing interests

The authors declare that they have no competing interests.

## Authors’ contributions

CS and KO carried out Western blotting studies. KS drafted the manuscript. YI, HM, KI, TT, and YK carried out evaluation of the samples and the immunoassays. KJT and TW performed the statistical analysis. KH carried out measurements of amino acids. KN and NM conceived of the study, and participated in its design and coordination and helped to draft the manuscript. All authors read and approved the final manuscript.

## Supplementary Material

Additional file 1 Relationships between age and expression levels of proteins. Click here for file

Additional file 2 Relationships between age and concentrations or ratio of glutamate and glutamine. Click here for file
