# Supplementary figures and images for: Enzymes in the glutamate-glutamine cycle in the anterior cingulate cortex in postmortem brain of subjects with autism
Source: Mol Autism. 2013 Mar 26;4:6. doi: 10.1186/2040-2392-4-6 (PMC3621600; doi:10.1186/2040-2392-4-6)

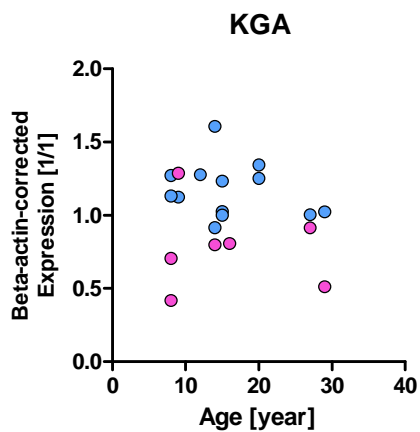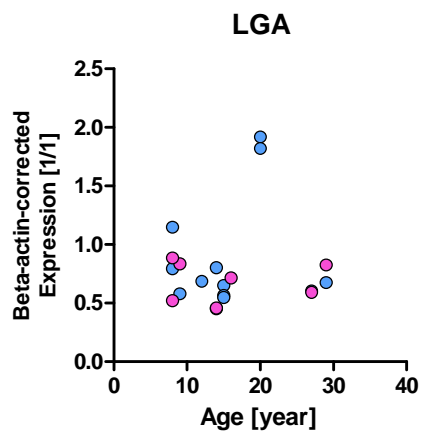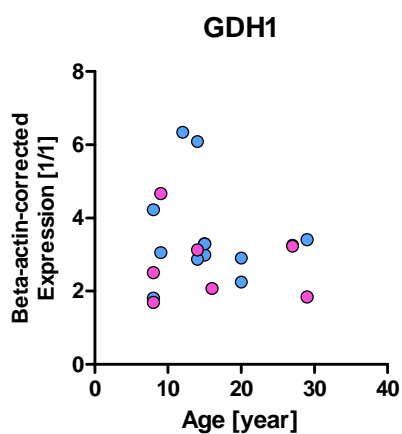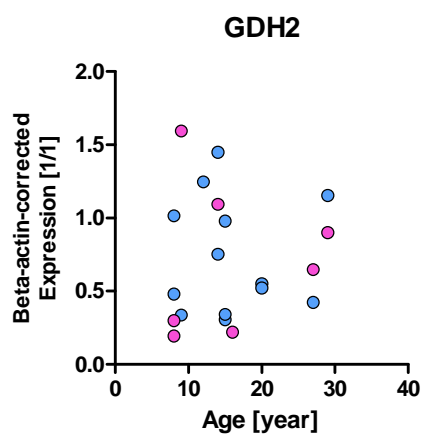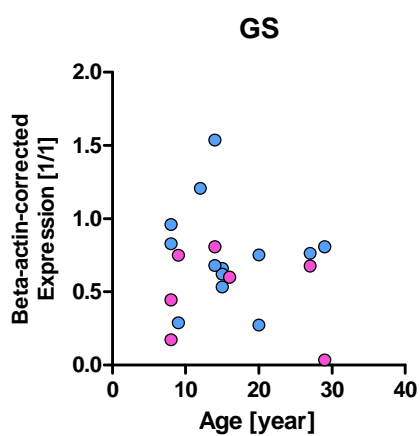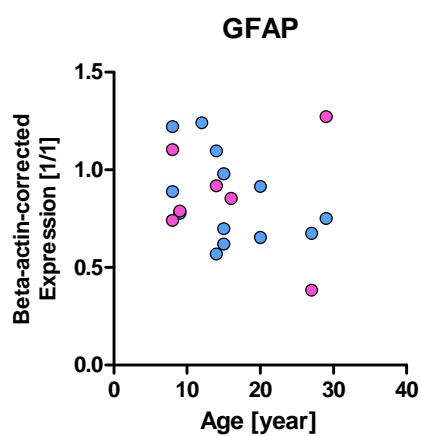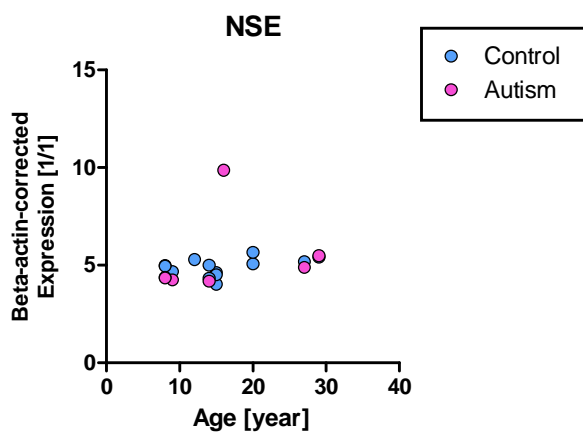

Supplement: Additional file 1 — Relationships between age and expression levels of proteins. [file 2040-2392-4-6-S1.pdf]

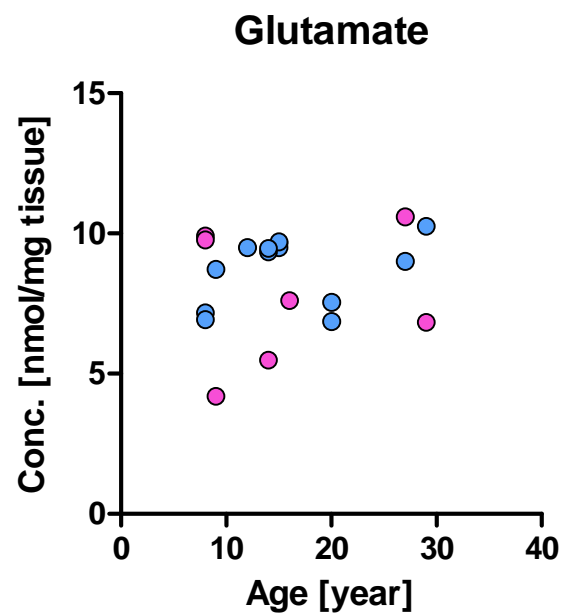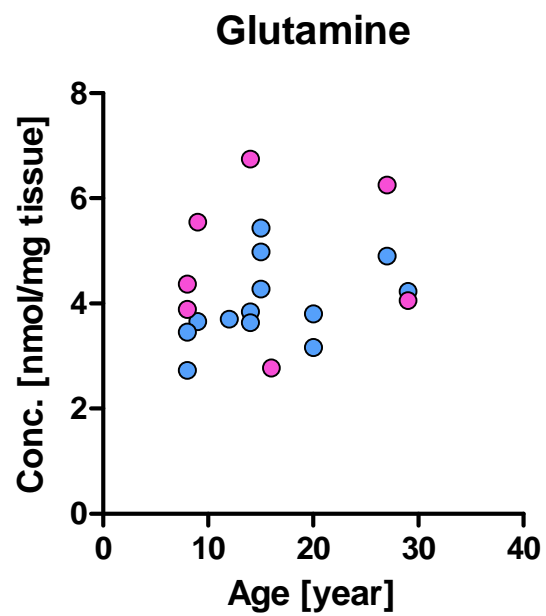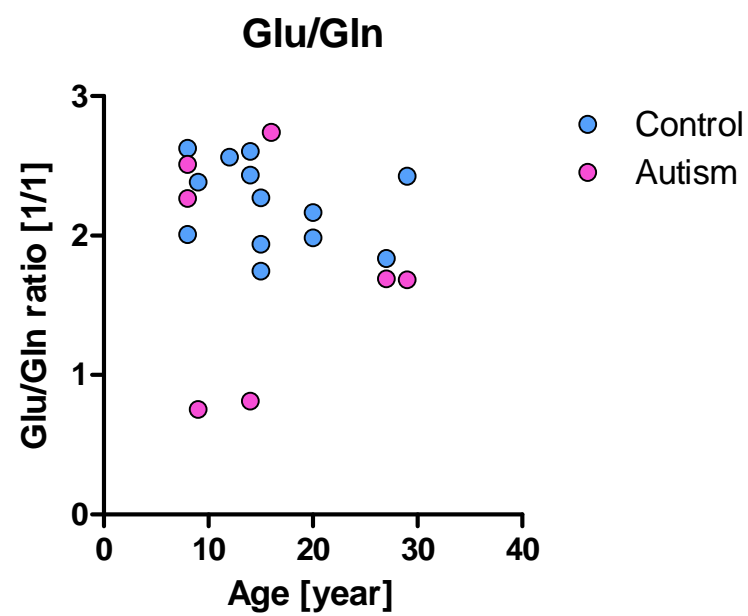

Supplement: Additional file 2 — Relationships between age and concentrations or ratio of glutamate and glutamine. [file 2040-2392-4-6-S2.pdf]
